# Pinnipeds orient and control their whiskers: a study on Pacific walrus, California sea lion and Harbor seal

**DOI:** 10.1007/s00359-020-01408-8

**Published:** 2020-02-20

**Authors:** Alyxandra O. Milne, Catherine Smith, Llwyd D. Orton, Matthew S. Sullivan, Robyn A. Grant

**Affiliations:** 1 Department of Natural Sciences, Manchester Metropolitan University, Manchester, M1 5GD UK; 2 Events Team, Blackpool Zoo, Blackpool, FY3 8PP UK; 3 RSPCA Stapeley Grange, Nantwich, CW5 7JW UK; 4 Department of Life Sciences, Manchester Metropolitan University, Manchester, M1 5GD UK

**Keywords:** Vibrissae, Touch sensing, Foraging ecology, Marine mammals, Movement analysis

## Abstract

Whisker touch is an active sensory system. Previous studies in Pinnipeds have adopted relatively stationary tasks to judge tactile sensitivity, which may not accurately promote natural whisker movements and behaviours. This study developed a novel feeding task, termed *fish sweeping* to encourage whisker movements. Head and whisker movements were tracked from video footage in Harbor seal (*Phoca vitulina*), California sea lion (*Zalophus californianus*) and Pacific walrus (*Odobenus rosmarus divergens*). All species oriented their head towards the moving fish target and moved their whiskers during the task. Some species also engaged in whisker control behaviours, including head-turning asymmetry in the Pacific walrus, and contact-induced asymmetry in the Pacific walrus and California sea lion: behaviours that have only previously been observed in terrestrial mammals. This study confirms that Pinnipeds should be thought of as whisker specialists, and that whisker control (movement and positioning) is an important aspect of touch sensing in these animals, especially in sea lions and walruses. That the California sea lion controls whisker movement in relation to an object, and also had large values of whisker amplitude, spread and asymmetry, suggests that California sea lions are a promising model with which to further explore active touch sensing.

**Electronic supplementary material:**

The online version of this article (10.1007/s00359-020-01408-8) contains supplementary material, which is available to authorized users.

## Introduction

Active sensing describes the purposive and information-seeking movement of sensors to improve the quality and quantity of the sensory information they obtain (Grant et al. 2009; Prescott et al. 2011). Active sensing in animals is perhaps most prevalent in whisker touch sensing (Prescott et al. 2011), with rodents, shrews and Pinnipeds often identified as *whisker specialists* (Grant and Arkley 2016). These animals actively control their whiskers to guide locomotion, exploration, foraging and navigation (Grant et al. 2009, 2018a; Grant and Arkley 2016). Pinniped whiskers, in particular, have been studied, due to their prominence, high sensitivity (Hyvärinen and Katajisto 1984; Hyvärinen 1989; Dehnhardt et al. 1998; Mauck et al. 2000; Marshall et al. 2006; Hyvärinen et al. 2009; Erdsack et al. 2014; McGovern et al. 2014) and their ability to be moved using a network of voluntary muscles (Berta et al. 2005). Indeed, Pinniped whiskers are capable of the tactile discrimination of object textures, shapes and sizes to a similar sensitivity as human fingertips (Dykes 1975; Murphy et al. 2015) and can also detect fine-scale water movements, termed hydrodynamic sensing (Dehnhardt et al. 2001; Wieskotten et al. 2010a, b; Krüger et al. 2018).

Studies in Pinnipeds, including California sea lion (*Zalophus californianus*), Harbor seal (*Phoca vitulina*) and Pacific walrus (*Odobenus rosmarus divergens*) have mainly focussed on static discrimination tasks to study whisker sensitivity (Dehnhardt 1990; Kastelein et al. 1990; Dehnhardt and Kaminski 1995; Dehnhardt and Dücker 1996; Dehnhardt et al. 1997; Grant et al. 2013). During these tasks, it has been described that the animal orients its head to the stimuli, such that it can be touched by vibrissae on both sides of the head, and that the animal may move its whiskers against or over the stimuli (Kastelein et al. 1990; Dehnhardt 1994; Dehnhardt and Dücker 1996). Pinnipeds can lie their vibrissae back against their muzzle or they can be protracted forward, especially during object contact (Dehnhardt et al. 2001; Gläser et al. 2010). Pinnipeds utilise lateral head movements during object exploration to position their whiskers (Kastelein and Van Gaalen 1988; Dehnhardt 1994; Dehnhardt and Kaminski 1995; Dehnhardt et al. 2001; Miersch et al. 2011). Therefore, it has been suggested that head positioning, rather than whisker control, drives the placement of whisker positions on to sensory stimuli in Pinnipeds (Dehnhardt 1994; Grant et al. 2013). Indeed, while vibrissal touch is often thought of as an active sensory system (Prescott et al. 2011; Grant and Arkley 2016), previous studies in Pinnipeds have not encouraged or measured, whisker movements.

The exception to this is a study by Milne and Grant (2014), who developed a novel, dynamic sensorimotor task to promote whisker movements. They demonstrated that whisker movements were important during a ball-balancing task in California sea lions; specifically, whisker movements occurred much sooner than the head, in response to movements of the ball, and were employed to help sense and control the ball (Milne and Grant 2014). However, since the number, arrangement, size, stiffness and structure of the whiskers vary significantly between Pinnipeds (Ling 1977; Watkins and Wartzok 1985), these observations might not hold true for other species, such as walrus and seal. In addition, not all Pinnipeds are able to ball balance; therefore, another behavioural task is needed to allow for comparisons of naturalistic whisker and head movements across a variety of Pinniped species. The aim of this study was to provide a description of whisker movements and control in Pinnipeds, by comparing and quantifying whisker movements in California sea lion, Harbor seal and Pacific walrus. A novel, feeding task was designed to promote whisker movements in these three species, and enable fair comparisons between them.

## Methods

### Animals

One species was selected across each of the three Pinniped families: Otariid, Phocid and Odobenid. They were: California sea lion (*Zalophus californianus*), Harbor seal (*Phoca vitulina*) and Pacific walrus (*Odobenus rosmarus divergens*). The five California sea lions used in this study were housed at the Active Oceans area in Blackpool Zoo, UK: Gina (16 years old), Anya (12 years old), Lo (15 years old), Gala (16 years old) and Fillipa (20 years old). Harbor seals were housed at Rhyl SeaQuarium, UK. The three animals used in this study were Wanda (22 years old, with cataracts on both eyes), Ina (16 years old) and Pamina (14 years old). Three Pacific walrus were used: Olga (35 years old, considered completely blind), Rossita (22 years old) and Olivia (9 years old) that were all housed at the Dolphinarium Harderwijk, The Netherlands. Only female animals were used because there were multiple adult females housed within each collection who were all trained for displays. Indeed, all animals were chosen for their trainability, access and availability. As the task was to film the Pinnipeds feeding, none of the animals needed to be trained any new behaviours for this study. The animals were not blindfolded for this study to give ethologically relevant values of whisker movements (in line with the procedures in Milne and Grant 2014), since in the wild whisker movements will never occur in the absence of vision, unless the animal is blind or they are hunting on a particularly dark night or in murky water. Blindfolding the animal may also increase their reliance on whisker touch, causing increases in whisker amplitude that would not be representative of usual whisker movements (Arkley et al. 2014; Grant et al. 2018b). In addition, these animals were all zoo animals, and not trained for research activities; therefore, blindfolding was considered to expose them to unnecessary suffering and stress.

### Experimental procedures

**Fig. 1:**
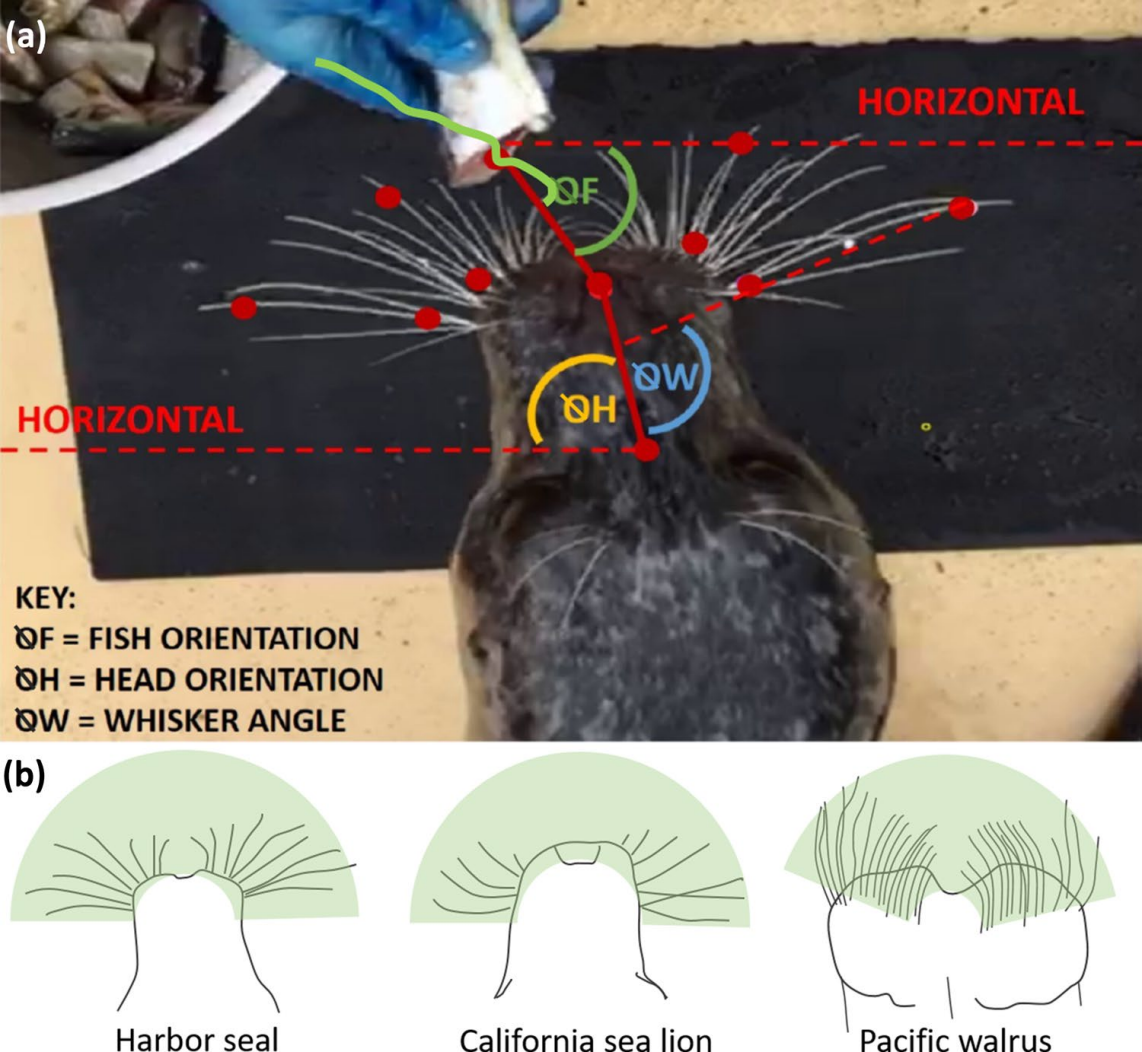
Example methods figure with fish positioning and tracking. **a** Video head and whisker tracking with angular measurements for the fish (green), head (yellow) and whisker (blue) angles. Demonstrated here on a video still of a Harbor seal. The fish tracking, before and after the capture of this video still, can be visualised using the green line, moving rightwards from the bucket towards the seal. The red dots correspond to the tracked points of the head, fish and whiskers. **b** Example positions (in green) where the fish could be positioned around the head. Note that the smaller area of possible fish locations in the walrus, due to its more forward-positioned whiskers. The walrus could not detect the fish outside of this location

Two behavioural feeding tasks were designed to induce whisker movements. Firstly, *fish catching*, where fish were thrown to the animals, in much the same way that the animals are usually fed, but with deviations in projection such that individuals had to move their head to intercept the fish (similar to Fig. 6 in Milne and Grant 2014). However, during a pilot study, Pacific walruses were unable to catch the fish; therefore another task was designed, termed *fish sweeping*. Fish sweeping involved a trainer moving fish over the whiskers of the animals from one side to the other. Since the animals have all been trained from a young age to take food from the hand, the animals did not need to undergo additional training. They have also been instructed not to snatch food from a young age; therefore, all individuals gently intercepted the fish and took it from the trainer. Any instances of snatching would have led to the termination of the session, although this did not occur in any instance. The trainer would sweep the fish over the animal’s whisker field, and the animal would follow and intercept the fish when it was ready, which would take 0.5–1.2 s in California sea lions, 0.4–0.9 s in Harbor seals and 1.0–1.1 s in Pacific walruses. This task could be successfully conducted on all species, although the walruses, being less mobile, could only follow smaller and slower fish sweeps. This meant that fish sweeping in the California sea lion and Harbor seal was conducted from 0° to 180° at speeds of 0°–4°/s over the whole pad, but only over 60°–120° at speeds of 0°–0.3°/s in the Pacific walrus (see Fig. 1 for fish orientation angle (θF), and Fig. 3 for the range of fish orientations in each species). Outside of this range, the walruses were unable to follow the fish and would lose interest in it. Differences in fish movement were controlled for in statistical analyses (see “Statistical considerations” below, and Supplementary Materials 1 and 2 for more information). A variety of fish, including mackerel (*Scomber scombrus*), herring (*Clupea harengus*), capelin (*Mallotus villosus*) or sprats (*Sprattus *sp.) were given to the Harbor seals and California sea lions during the task. For the Pacific walruses, herring (*Clupea harengus*), sprats (*Sprattus *sp.) or squid (*Loligo opalescens*) were given. Fish were included as part of the animals’ daily food amount and food was not restricted.

Testing was conducted in February and March 2018 when none of the animals were in moult (moult occurs in the summer months for all species). Trials occurred at varying schedules throughout the day, limited to 10 min, three to five times a day. Trials were carried out in training areas at each of the institutions, which were already familiar to the animals. To enable the whiskers to be seen clearly, the experiment was conducted in the shade or against a dark background (such as a mat, Fig. 1) where possible. Display stations were positioned so the California sea lions could elevate their forelimbs easily during the task. Harbor seals were placed in a training pool or on the land over a black mat and the Pacific walruses were positioned on the ground standing on their forelimbs, with their hind limbs relaxed on the floor. All animals were free to move their head and body during the sweeping task. However, since the fish movement was relatively small, the animals always kept their flippers and body on the floor or training station throughout the task, so only their head and whiskers moved towards the fish. All animals could leave their training areas at any time. During the trials, the same trainers were present each time the task took place. Trainer 1 would move a fish past the animal’s head in a sweeping motion, allowing the animal to take it. Trainer 2 filmed from above and made sure the head and whiskers were in view for the full sweep. Whisker filming for the Harbor seal and the California sea lion was conducted using a waterproof GE DV1 Pocket Digital Camcorder (HD 1080p, 30 fps). For the Pacific walrus, filming was completed using a handheld iPhone camera (30 fps). Positive reinforcement was used to increase the animal’s attention and maintain performance. The number of times this happened in a session varied allowing multiple trials to be obtained per video (Milne and Grant 2014).

### Video selection and analysis

All video clips were examined to ensure that videos selected would show the following: (i) the whiskers and head were all in view of the camera throughout the clip, (ii) the head appeared to be flat to the floor, therefore not having any instances of extreme pitch or roll, which would impact 2D tracking, and (iii) all the whiskers were clearly lit and visible. Each video was tracked manually using an open source tracker, the ‘Manual Whisker Annotator’ (MWA) (Hewitt et al. 2016). Two whiskers on each side of the face were tracked along with the nose and a head point, in between the eyes (Fig. 1).

Whiskers selected for tracking were the second most rostral and second most caudal whisker on each side of the muzzle (as per Milne and Grant 2014). Two points were tracked on each whisker: one point close to the base of the whisker shaft and one point over two-thirds along the whisker shaft (Fig. 1). Tracking was started once the fish had entered the shot from Trainer 1 and continued until the frame before the animal opened its mouth to eat the fish. After reviewing all the videos, a total of 48 clips were used for tracking, with data from each individual: 22 clips for the Harbor seals (Wanda: 10, Ina: 7, Pamina: 5), 21 clips for the California sea lions (Gina: 6, Anya: 6, Lo: 5, Gala: 2, Fillipa: 2), and 5 clips for the Pacific walruses (Rossita: 1, Olga: 2, Olivia: 2). Lower values were observed for the walruses. Keeper access to the walruses was more restricted; therefore, it was hard to line up all the video shots. In addition, the walruses have large heads and many whiskers, and it was challenging to keep all the whiskers in shot throughout the clip. From the videos, fish and head orientation and whisker angle was calculated (Fig. 1) Whisker variables were also calculated, including whisker offset, whisker asymmetry, whisker amplitude and whisker spread (Milne and Grant 2014). All variable definitions can be found in Table 1.

**Table 1:** Measurements and whisker variables: Whisker variables used with definitions

Measure variables (in degrees)	Definition
Fish orientation	The angle between the fish and nose point, calculated as the angle from each fish point to the nose tip, from the horizontal. Per frame measure
Head orientation	The angle between the head and nose point, calculated as the angle from each head point to the nose tip, from the horizontal. Per frame measure
Whisker angle	The angle between the whisker and the midline of the head. Per frame measure
Whisker offset	The mean whisker angle calculated by averaging all the whisker angular positions in each frame. Per clip measure, averaged from all tracked whiskers
Whisker amplitude	Calculated as the difference between the maximum and minimum whisker angular positions. Per clip measure, averaged over all tracked whiskers
Whisker asymmetry	The difference between the left whisker angular positions and the right (left minus right). Per clip measure, averaged for the front and back whiskers, or per clip measure, averaged for the front and back whiskers
Whisker spread	Calculated as the angular difference between the front and back whiskers. Per clip measure, averaged for the two sides

### Statistical considerations

**Fig. 2:**
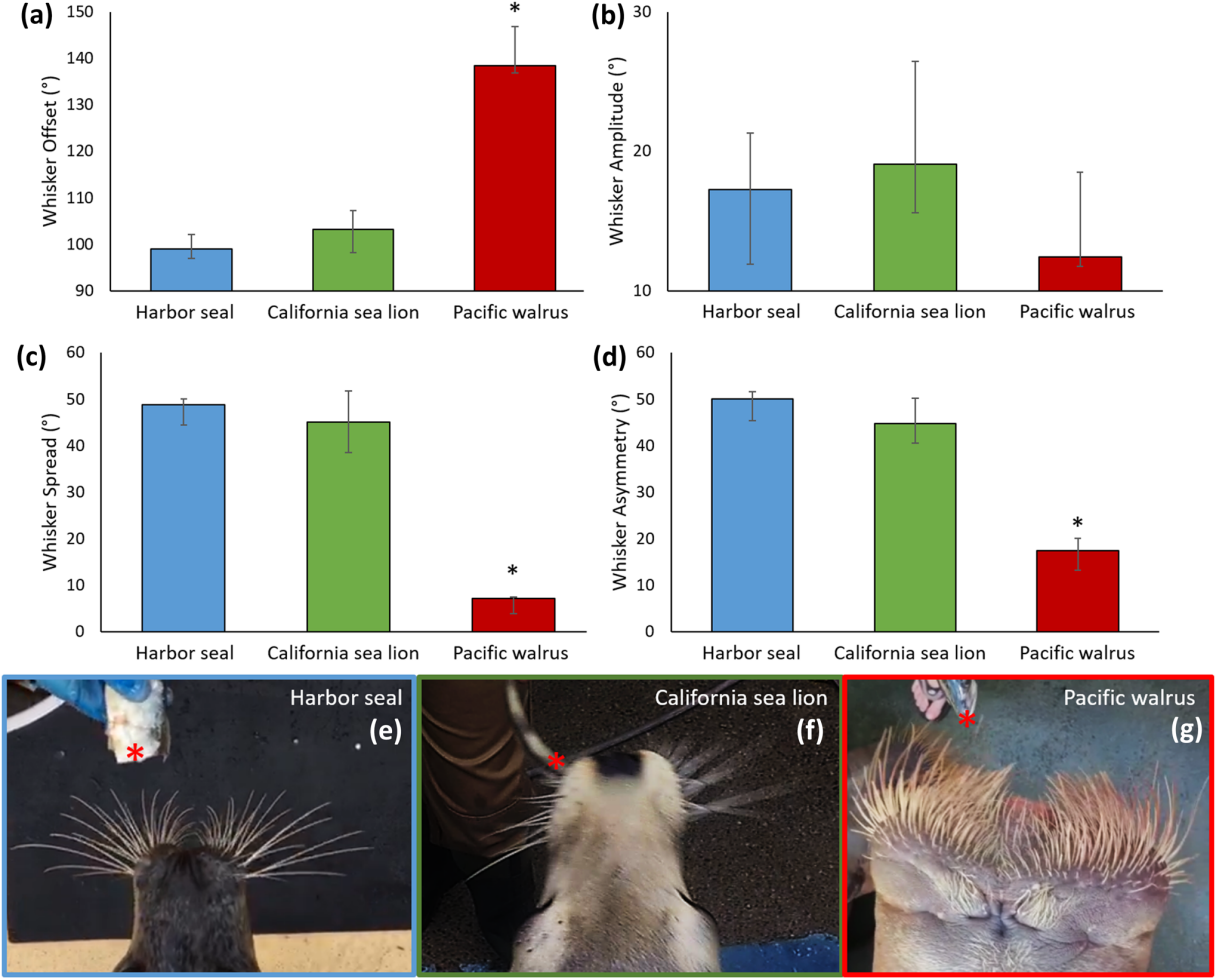
Pinniped whisker positions and movements: **a** Whisker offset values show that Pacific walruses have higher offset values; **b** Whisker amplitude not significantly different between the three species; **c** Whisker spread is lowest in the Pacific walrus; **d** Whisker asymmetry is smallest for the Pacific walrus. All graphs show median values in degrees with error bars indicating upper and lower interquartile ranges. Asterisks (*) show significant differences Mann–Whitney *U* post hoc (*p* < 0.05). Data corresponds to 48 clips: 21 California sea lion clips (including data from 5 individuals), 22 Harbor seal clips (including data from 3 individuals) and 5 Pacific walrus clips (including data from 3 individuals). **e**–**g** show example video stills to summarise the findings in the graphs, for Harbor seal, California sea lion and Pacific walrus. Red asterisk corresponds to the leading edge of the fish. Whiskers of the California sea lion are slightly blurred, owing to their fast movements (**f**)

**Fig. 3:**
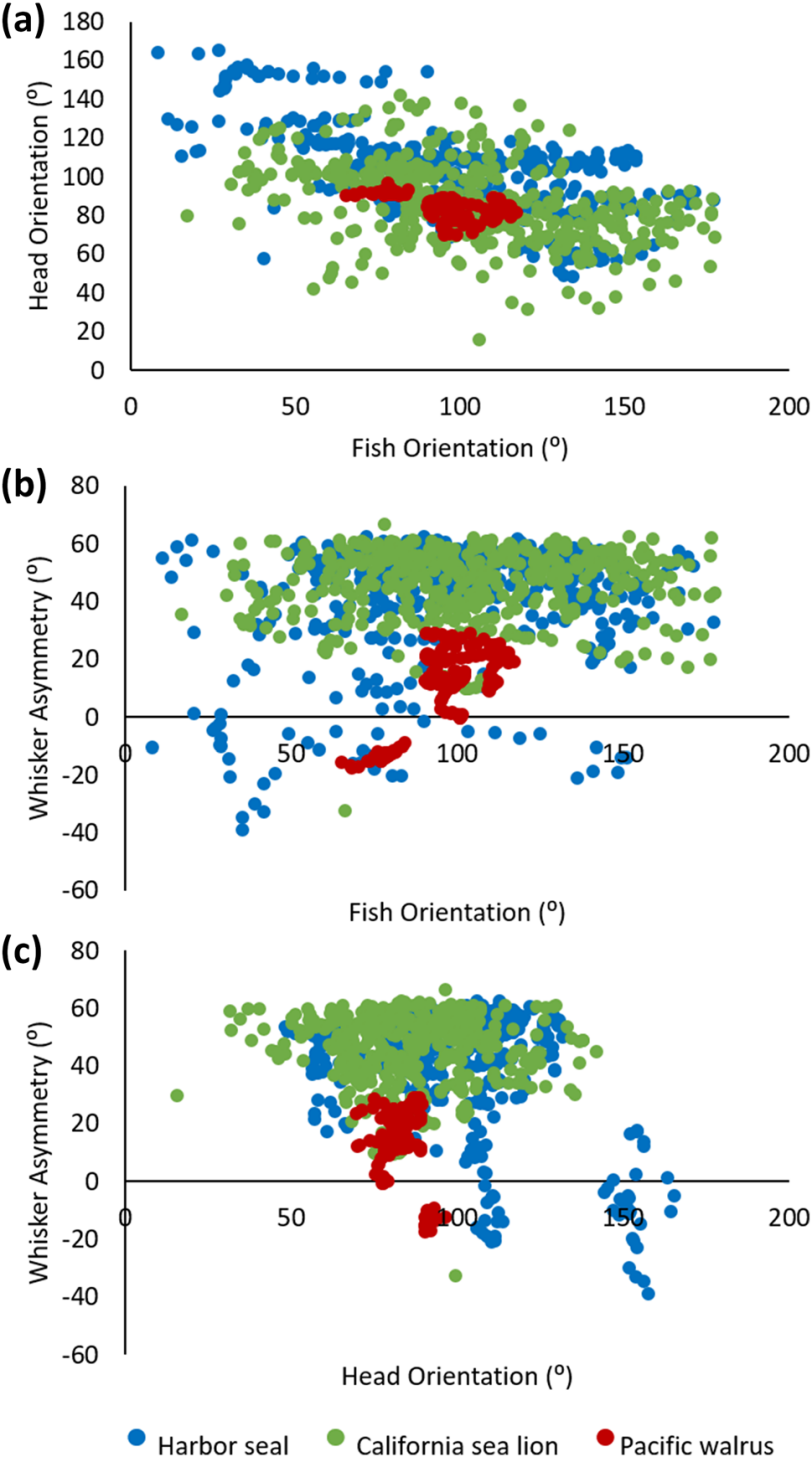
Head, fish and whisker angles in response to fish sweeping. Scattergrams of per-frame angles across all tracked videos for: **a** Fish Orientation vs. Head Orientation, **b** Fish Orientation vs. Whisker Asymmetry, **c** Head Orientation vs. Whisker Asymmetry. Head orientation is correlated to the fish orientation in all species (Spearman’s rank Correlation: *p* < 0.05), in (**a**). Fish orientation and whisker asymmetry is correlated in California sea lion and Pacific walrus (Spearman’s rank Correlation: *p* < 0.05), but not in Harbor seal in (**b**). Whisker asymmetry and head orientation is correlated in Pacific walrus in (**c**) (Spearman’s rank Correlation: *p* < 0.05), but not in California sea lion or Harbor seal. Data corresponds to 48 clips: 21 California sea lion clips (including data from 5 individuals), 22 Harbor seal clips (including data from 3 individuals) and 5 Pacific walrus clips (including data from 3 individuals)

All 48 clips, with data from all individuals, were used for data analysis and presented in the results figures in Figs. 2 and 3. Per-clip whisker movement and position measures (whisker offset, amplitude, asymmetry and spread) were compared between the three species, using a one way Kruskal–Wallis test, with Mann–Whitney *U* tests employed to test for post hoc pairwise comparisons. Per-clip whisker position and movement variables were re-tested (using the Kruskal–Wallis test with Mann–Whitney *U* pairwise comparisons) controlling for the movement of the fish by: (i) only including video frames with fish orientations of 65°–120° and (ii) only including video frames with fish orientations of 65°–120° and fish speeds of 0°–0.3°/s (Supplementary Material 1). A Spearman’s rank test was used to test for correlations of the per-frame head orientation, fish orientation and whisker asymmetry variables. Per-frame variables were re-tested controlling for the movement of the fish by: (i) only including video frames with fish orientations of 65°–120°; (ii) only including video frames with fish orientations of 65°–120° and controlling for fish speed using partial correlations; (iii) only including video frames with fish orientations of 65°–120° and fish speeds of 0°–0.3°/s (Supplementary Material 2). Non-parametric tests were used since the data were not normally distributed. Bar charts present median values with error bars indicating upper and lower interquartile ranges.

## Results

### Whisker movements and position

Figure 2 shows that there were significant species differences in measurements of whisker positions and movement, including offset (Kruskal–Wallis: *χ*^2^ = 15.834, df = 2, *p* ≤ 0.001), spread (Kruskal–Wallis *χ*^2^ = 14.083, df = 2, *p* = 0.001) and asymmetry (Kruskal–Wallis *χ*^2^ = 13.831, df = 2, *p* = 0.001). Amplitude did not significantly differ between the three species (Kruskal–Wallis *χ*^2^ = 3.511, df = 2, *p* = 0.173). These results were also confirmed when fish orientation and speed were controlled for (Supplementary Material 1). Post hoc tests confirm that the largest offset values were observed in the Pacific walrus (Fig. 2a), compared to both the Harbor seal and California sea lion. Indeed, the whiskers of Pacific walruses are naturally positioned very far forward on their face, which probably accounts for these high offset values (Fig. 2g).

The largest spread values were seen in Harbor seals and California sea lions (which was also confirmed with post hoc tests) (Fig. 2c). This can be seen in Fig. 2e, as the Harbor seal rostral whiskers were more forward than the caudal whiskers, causing the spreading out of the whiskers. California sea lion whiskers were also more spread out that the Pacific walrus whiskers, (Fig. 2f), as the Pacific walruses’ whiskers were densely packed together with similar angles (Fig. 2g). As well as moving all their whiskers forward and spreading them out, whiskers between the left and right side can also be moved independently, causing asymmetry in the whisker angles between the two sides (Fig. 2c). Post hoc tests confirm that the smallest asymmetry values were observed in the Pacific walrus (Fig. 2d), compared to both the Harbor seal and California sea lion. This can clearly be seen by comparing the video stills of the Pacific walrus (Fig. 2g) to the California sea lion (Fig. 2f), where the sea lion whiskers on the left side are retracted back, while the whiskers on the right side are protracted forward.

During the sweeping task, all species moved their whiskers at similar amplitudes (Fig. 2b). Although not statistically significant, California sea lions moved their whiskers more (with higher amplitudes) than the Harbor seals, and the Pacific walruses had the lowest amplitude. This is illustrated in Fig. 2f, where the whiskers of the California sea lion are slightly blurred, owing to their fast movements.

### Whisker and head orienting

**Fig. 4:**
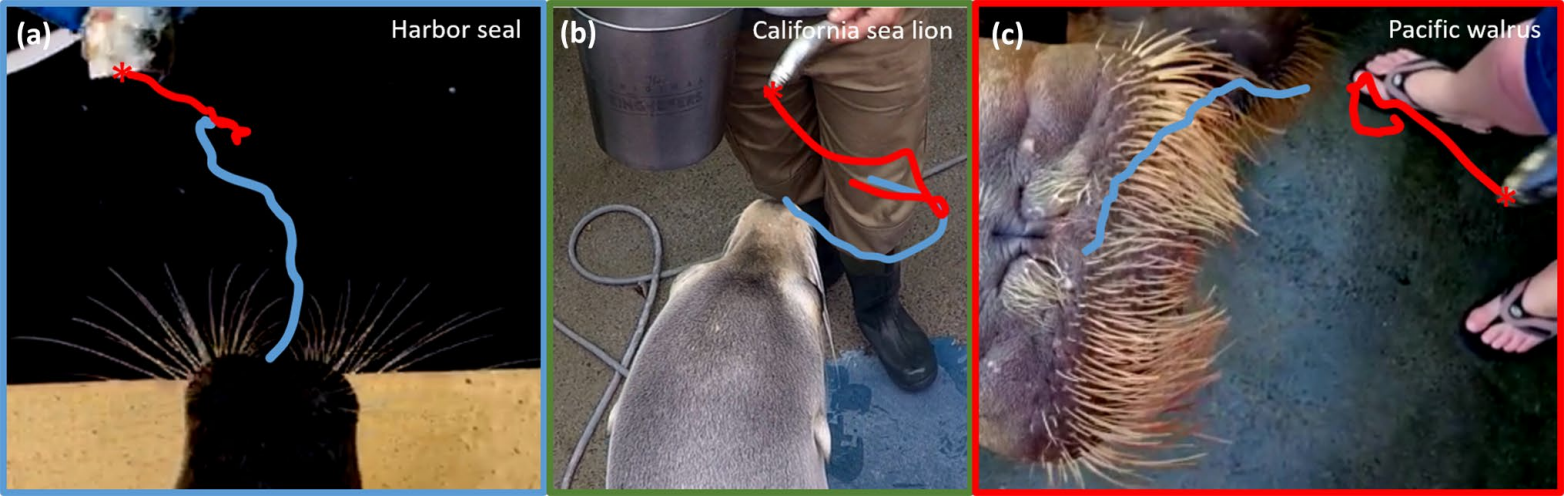
Example video stills with overlaid nose (in blue) and fish (in red) tracking. Tracking every frame, over 25 frames (0.84 s) for Harbor seal (**a**) and Pacific walrus (**c**) and 15 frames (0.50 s) for California sea lion. The pictured video frame shows the start of the sequence (*t* = 0). Note that all three species move their head to follow and intercept the fish. Examples of California sea lion (**b**) and Pacific walrus (**c**) especially show the head precisely following the movement of the fish; from right to left to right in California sea lion, and bottom to top in the Pacific walrus

All species oriented their heads towards the sweeping fish. Therefore, there was a significant negative correlation between the fish orientation and head orientation in the Harbor seal (Spearman’s rank: *r* = − 0.466, *p* < 0.001), California sea lion, (Spearman’s rank: *r* = − 0.611, *p* < 0.001) and Pacific walrus (Spearman’s rank: *r* = − 0.498, *p* < 0.001) (Fig. 3a). These results were also confirmed when fish orientation and speed were controlled for (Supplementary Material 2). Orientation of the head towards the fish can be seen in the nose and fish tracked examples in Fig. 4, where the fish is moved in one direction, and the Harbor seal, California sea lion and Pacific walrus all move their heads to intercept the fish. In the California sea lion and Pacific walrus tracked example, the movement of the head precisely follows the movement of the fish; from bottom to top in the Pacific walrus (Fig. 4c), and left to right in the sea lion (Fig. 4b).

**Fig. 5:**
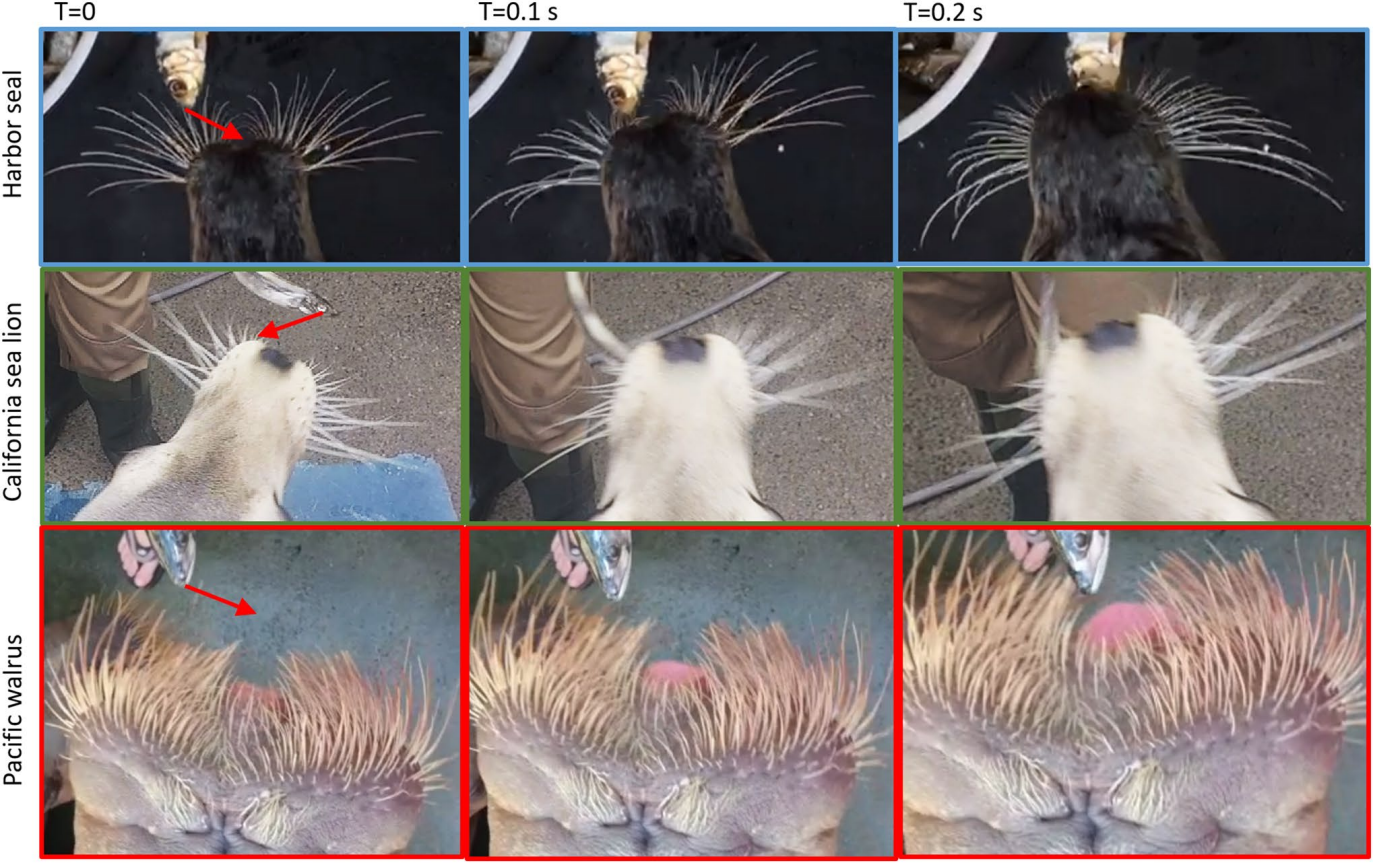
Example video stills during fish sweeping task for Harbor seal, California sea lion and Pacific walrus. Red arrow in the first panel corresponds to direction of the sweeping fish. The top panel shows a Harbor seal orienting their head leftwards towards the fish. As the head moves left the whiskers retract on the left-hand side (middle panel). The range of movement of the whiskers (amplitude) can also be seen in the Harbor seal, from protraction on the left, to retraction on the right. The middle panel shows a California sea lion orienting their head leftwards towards the fish. As the head is tilted towards the right in the left panel, the whiskers are symmetrical; however, as the fish is moved to the left in the middle panel, the head is central and the whiskers are asymmetric, with the left-hand whiskers more retracted than the right-hand whiskers. This demonstrates the whiskers orienting towards the fish in California sea lions and that the. The bottom panel shows a Pacific walrus orienting their head leftwards towards the fish. As the head is tilted towards the right in the left panel, the whiskers are more protracted on the left-hand side, when the head is more central to the fish, the whiskers are more symmetric (right panel), demonstrating the coupling of whisker and head movements. Whisker movements of the walrus are quite small compared to the other species

California sea lions and Pacific walruses also oriented their whiskers towards the sweeping fish (Fig. 3b). There was a significant positive relationship between whisker asymmetry and fish orientation in the California sea lions, (Spearman’s rank: *r* = 0.136, *p* = 0.006) and Pacific walruses (Spearman’s rank: *r* = 0.482, *p* < 0.001), but not in the Harbor seals, (Spearman’s rank: *r* = 0.037, *p* = 0.460). These results were also confirmed when fish orientation and speed were controlled for (Supplementary Material 2). The Pacific walruses did not move their whiskers much, so it is hard to see whisker asymmetry towards the fish in Fig. 5. However, in the left panel, the whiskers are more asymmetric (with the left whisker protracted more forward than the right whiskers) when the fish is on the left of the face, and more symmetric when the fish is more central (right panel). The California sea lion example is very clear (middle panel). As the fish moves left (from the left to middle panel), the California sea lion whiskers in the middle panel were more protracted on the right and further back on the left (Fig. 5, middle panel), orienting towards the moving fish.

Whisker asymmetry and head orientation were significantly correlated in the Pacific walruses (Spearman’s rank: *r* = − 0.260, *p* = 0.001) and not in the Harbor seals (Spearman’s rank: *r* = 0.280, *p* = 0.280). These results were also confirmed when fish orientation and speed were controlled for (Supplementary Material 2). The California sea lions whisker asymmetry and head orientation were significantly correlated when using the whole dataset (Spearman’s rank: *r* = − 0.162, *p* = 0.001), although not when fish orientation and speed are controlled for (Supplementary Material 2). This can be seen in the video stills in Fig. 5. For example, in the Pacific walrus example, in the middle panel (*t* = 0.1 s, Fig. 5), the head is oriented more to the right and the whiskers on the left are protracted slightly more than the whiskers on the right. In the Harbor seal example (top panel), the whiskers are fairly symmetrical, despite the head being turned slightly to the left (*t* = 0.1 s, Fig. 5). However, in the California sea lion (*t* = 0.1 and 0.2 s, Fig. 5), large whisker asymmetry could occur in the absence of head rotations, when the head is central.

## Discussion

This study is the first to measure whisker movements in a number of Pinniped species. All Pinniped species tested here positioned their whiskers towards the fish stimulus by orienting their head (Fig. 3a). While head movements grossly positioned the whiskers, all species also moved their whiskers during the task (Fig. 2b). This confirms that whisker movements and positioning are controlled during a fish sweeping task in Pinnipeds. Whisker movements enable rapid sampling of environments during object and spatial exploration (Knutsen 2015), which boosts the quality and quantity of sensory information. Animals that make whisker movements are also thought to have higher tactile sensory acuity than those without whisker movements (Muchlinski et al. 2020), and Pinnipeds do have extremely sensitive whiskers (Rice et al. 1986; Hyvärinen 1989).

### Whisker control behaviours

In many terrestrial whisker specialists, whiskers often move ahead of a head rotation to scan the area that the head is moving into (Towal and Hartmann 2006); this is termed head-turning asymmetry (Mitchinson et al. 2011; Grant et al. 2012). Head-turning asymmetry has previously been documented in rodents (Towal and Hartmann 2006; Mitchinson et al. 2011) and marsupials (Mitchinson et al. 2011). While all the Pinniped species tested here oriented their head towards the fish, in Pacific walrus their whisker asymmetry was also correlated to head orientation, which provides evidence of head-turning asymmetry in these species. Whisker asymmetry has not previously been documented in walrus, which is not surprising as their whisker movements are small (Fig. 2c, b).

As well as head-turning asymmetry, many terrestrial mammals also engage in contact-induced asymmetry (Mitchinson et al. 2011), where whiskers are positioned asymmetrically towards an object. Contact-induced asymmetry has been previously observed in rodents and insectivores (Mitchinson et al. 2011; Grant et al. 2018a). Both the Pacific walrus and the California sea lion oriented their whiskers towards the fish, with asymmetry correlating with fish orientation. Contact-induced asymmetry is likely to increase the number of whisker contacts to improve tactile sampling (Kastelein et al. 1990; Dehnhardt 1994; Mitchinson et al. 2007, 2011; Grant et al 2013; Milne and Grant 2014). This might indicate a reliance on touch sensing in the Pacific walrus and California sea lion when targets are in close proximity; indeed, whiskers have been previously documented as proximal sensors in both rodents (Towal and Hartmann 2006) and cats (Gottschaldt et al. 1974). That the California sea lions control their whisker positions in relation to an object, and also had large values of whisker amplitude, spread and asymmetry (Fig. 2), suggests that California sea lions, in particular, are a promising model from which to further explore active touch sensing in Pinnipeds.

### Whisker positions and movements

All the Pinniped species tested here moved their whiskers with similar amplitudes (median amplitudes of 19° in California sea lions, 17° in Harbor seal and 12° in Pacific walrus; Fig. 2b). Whisker amplitudes in terrestrial mammals tend to be higher than this, including rodents such as European dormice (*Muscardinus avellanarius*) (38°), wood mouse (*Apodemus sylvaticus*) (36°) and brown rat (*Rattus norvegicus*) (44°) (Arkley et al. 2017; Grant et al. 2018a). It might be that whisker movements are reduced somewhat in marine mammals, due to the energetics of moving them underwater. On the whole, Pacific walrus whisker positions and movements were quite different from Harbor seals and California sea lions. They had large offset values and small values of spread and asymmetry (Fig. 2), which is mainly due to their whiskers being densely packed and more forward facing than the other species. However, whisker kinematics have also been shown to be closely related to facial musculature across a variety of mammals (Grant et al. 2014, 2017; Muchlinski et al. 2020), with animals that move their whiskers more having thick and regular intrinsic muscles (Grant et al. 2017; Muchlinski et al. 2020). The differences in whisker positions and movements between species studied here could, therefore, be explained by anatomical differences that may exist in the follicles, mechanoreceptors, nerve fibres and muscle architecture (Kastelein et al. 1990), as well as the variation in whisker shape and length. For example, California sea lions and Harbor seals, that moved their whiskers the most, may have larger, more regular intrinsic muscles than the Pacific walrus. However, more studies would be required to fully characterise and address these anatomical differences.

### Implications

Differences in whisker positions and movements could also be associated with function, including feeding ecology, social interactions and guiding locomotion. The Pacific walrus forages for small, stationary prey underwater. It has many forward-oriented whiskers, which could enable the walrus to search for prey, using its whiskers much like a brush. Indeed, walruses have relatively small eyes positioned on the side of the head (Harington 2008) and when they search through a substrate for food, the water will become murky due to disturbance, making it challenging to use vision to find prey. Therefore, although touch is likely to be important, having moveable sensors might be less useful for the walrus. Larger whisker movements (amplitudes) and spread was observed in the California sea lions and Harbor seals. Both use a ‘pierce and grab’ feeding method on mobile prey items. They may move their whiskers to guide head rotations and touch during hunting, as well as for hydrodynamic sensing. Indeed, it has been noted that Harbor seals mainly always have their eyes shut during hunting and are likely to rely primarily on their whiskers (Marshall et al. 2014). Therefore, whisker movements might be important to guide foraging in Pinnipeds that hunt moving prey.

The interaction between other senses and whisker movements in Pinnipeds is not yet understood. That all the species oriented their heads towards the fish could indicate that the animals were following the fish using their vision or even by following the scent of the fish. However, the walruses were unable to follow the fish when it moved outside of their whisker field, despite their side-facing eyes that are probably still able to see it, suggesting that whisker touch is probably important in this task. It is difficult to compare our studies to other Pinniped haptic studies, since in most other studies the animal is usually blindfolded during a static discrimination task. However, a comparison of our Harbor seal whisker movements to those in a blindfolded, static shape discrimination task (Grant et al. 2013) shows that the values are very similar for amplitude (17°–19° in Grant et al. (2013), and 17° in this study, Fig. 2b). In rats (Arkley et al. 2014) and mice (Grant et al. 2018b), blind animals tend to have larger amplitude whisker movements, although we do not observe this in the Harbor seal example. In addition, in our study, one Pacific walrus was blind, and one Harbor seal had cataracts in both eyes, but these individuals did not have significantly different whisker movements or positions compared to other individuals of the same species (Supplementary Material 3).

Using this novel behavioural task has encouraged whisker movements in three species of Pinniped. Developing this study to include more species would allow comprehensive comparisons to be made between species, especially linking whisker behaviour with anatomy and function. Encouraging natural whisker movements in captive animals might also be considered a form of sensory and structural enrichment (Mackay 1981; Kastelein and Wiepkema 1988; de Azevedo et al. 2007; Clark et al. 2013); therefore, employing a feeding task of this nature might have positive implications for welfare. However, to better understand the extent of realistic whisker movements in Pinnipeds, it is imperative to study these animals in a more realistic way, especially during foraging, hunting and prey capture.

## Electronic supplementary material

Below is the link to the electronic supplementary material.

Supplementary file1 (PDF 623 kb)
