# Supplementary material for: Pinnipeds orient and control their whiskers: a study on Pacific walrus, California sea lion and Harbor seal
Source: J Comp Physiol A Neuroethol Sens Neural Behav Physiol. 2020 Feb 20;206(3):441–51. doi: 10.1007/s00359-020-01408-8 (PMC7192888; doi:10.1007/s00359-020-01408-8)
Supplement: Supplementary file 1 — Supplementary file1 (PDF 623 kb) [file 359_2020_1408_MOESM1_ESM.pdf]

Supplementary Material 1:

Pinniped whisker positions and movements, controlling for the movement of the fish.

Whisker position and movement variables were re-tested controlling for the movement of the fish by: i) only including video frames with fish orientations of 65-120° and ii) only including video frames with fish orientations of 65-120° and fish speeds of 0-0.3^o/s. The figures (Fig. S1 and S2) and the Kruskal Wallis statistical tests all confirm the results in the main manuscript document, that whisker amplitude does not significantly differ between the three species, but the Pacific walrus has larger whisker offset values and lower whisker spread and asymmetry, than the California sea lion and Harbor seal.

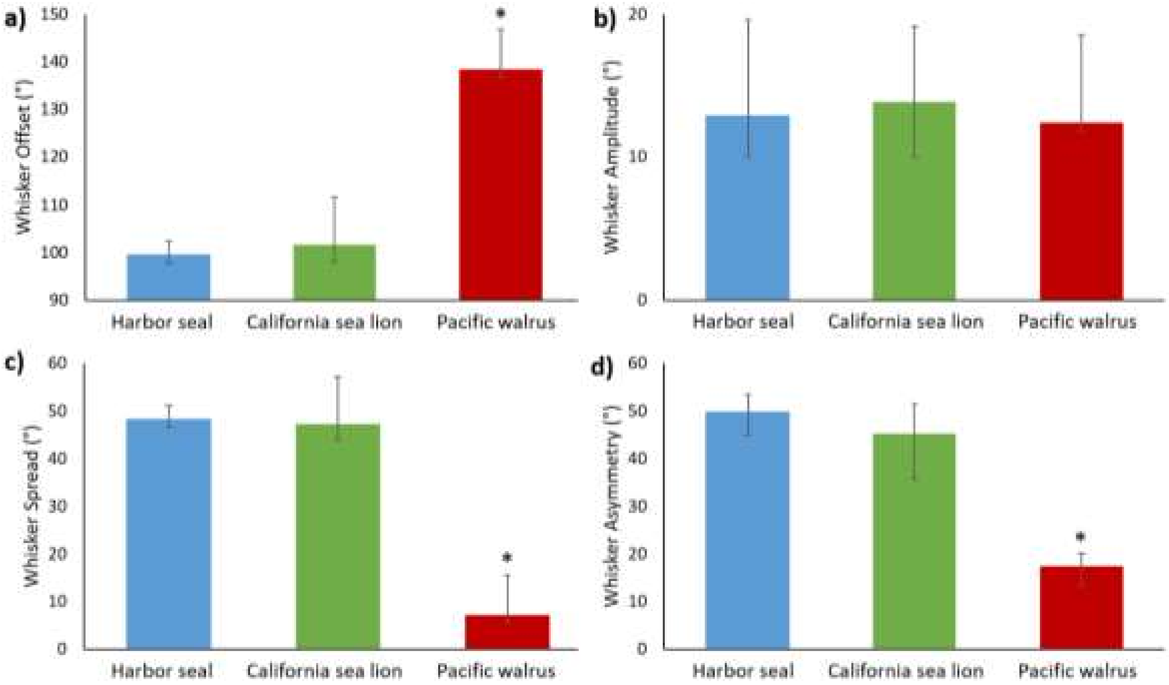

Fig. S1.1 Pinniped whisker positions and movements for fish orientations of 65-120°: (a) Whisker offset values show that Pacific walruses have higher offset values (Kruskal Wallis: $\chi 2$=14.386, df=2, p=0.001); (b) Whisker amplitude is not significantly different between the three species (Kruskal Wallis: $\chi 2$=0.128, df=2, p=0.938); (c) Whisker spread is lowest in the Pacific walrus (Kruskal Wallis: $\chi 2$=11.738, df=2, p=0.003); (d) Whisker asymmetry is smallest for the Pacific walrus (Kruskal Wallis: $\chi 2$=11.664, df=2, p=0.003). All graphs show median values in degrees with error bars indicating upper and lower interquartile ranges. Asterisks (*) show significant differences from Mann Whitney U post hoc tests ($p < 0.05$)

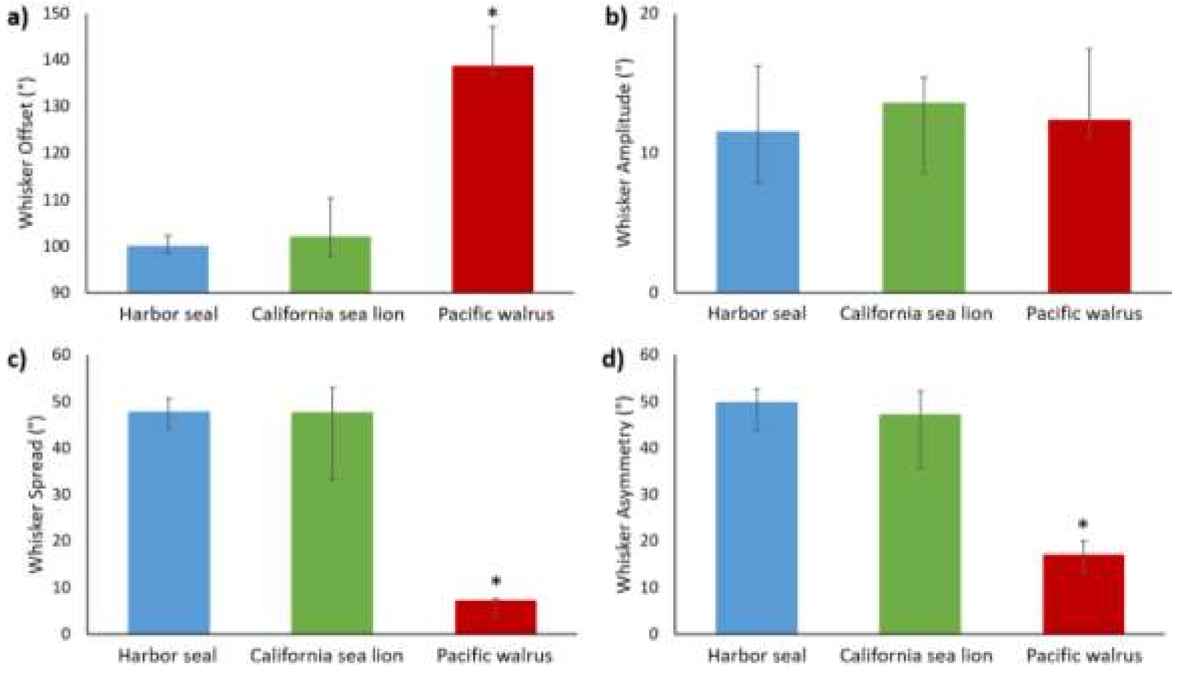

Fig. S1.2 Pinniped whisker positions and movements for fish orientations of 65-120° and fish speeds of 0-0.3$^{o/s}$: (a) Whisker offset values show that Pacific walruses have higher offset values (Kruskal Wallis: $\chi 2$=13.729, df=2, p=0.001); (b) Whisker amplitude is not significantly different between the three species (Kruskal Wallis: $\chi 2$=0.226, df=2, p=0.893); (c) Whisker spread is lowest in the Pacific walrus (Kruskal Wallis: $\chi 2$=11.932, df=2, p=0.003); (d) Whisker asymmetry is smallest for the Pacific walrus (Kruskal Wallis: $\chi 2$=10.617, df=2, p=0.005). All graphs show median values in degrees with error bars indicating upper and lower interquartile ranges. Asterisks (*) show significant differences from Mann Whitney U post hoc tests ($p < 0.05$)

Supplementary Material 2:

Head, fish and whisker angles in response to fish sweeping, controlling for the movement of the fish

Head, fish and whisker angles were re-tested controlling for the movement of the fish by: i) only including video frames with fish orientations of 65-120°; ii) only including video frames with fish orientations of 65-120° and controlling for fish speed using partial correlations; iii) only including video frames with fish orientations of 65-120° and fish speeds of 0-0.3^{o}/s. The figures (Fig. S3 and S4) and correlation tests all confirm the main findings in the manuscript that: i) fish orientation and head orientation are usually correlated in all three species; ii) that fish orientation and whisker asymmetry is correlated in California sea lion and Pacific walrus; iii) and head orientation and whisker asymmetry is not correlated in Harbor seal, but is correlated in Pacific walrus. A summary table (Table S1) below indicates the agreement of the correlation tests.

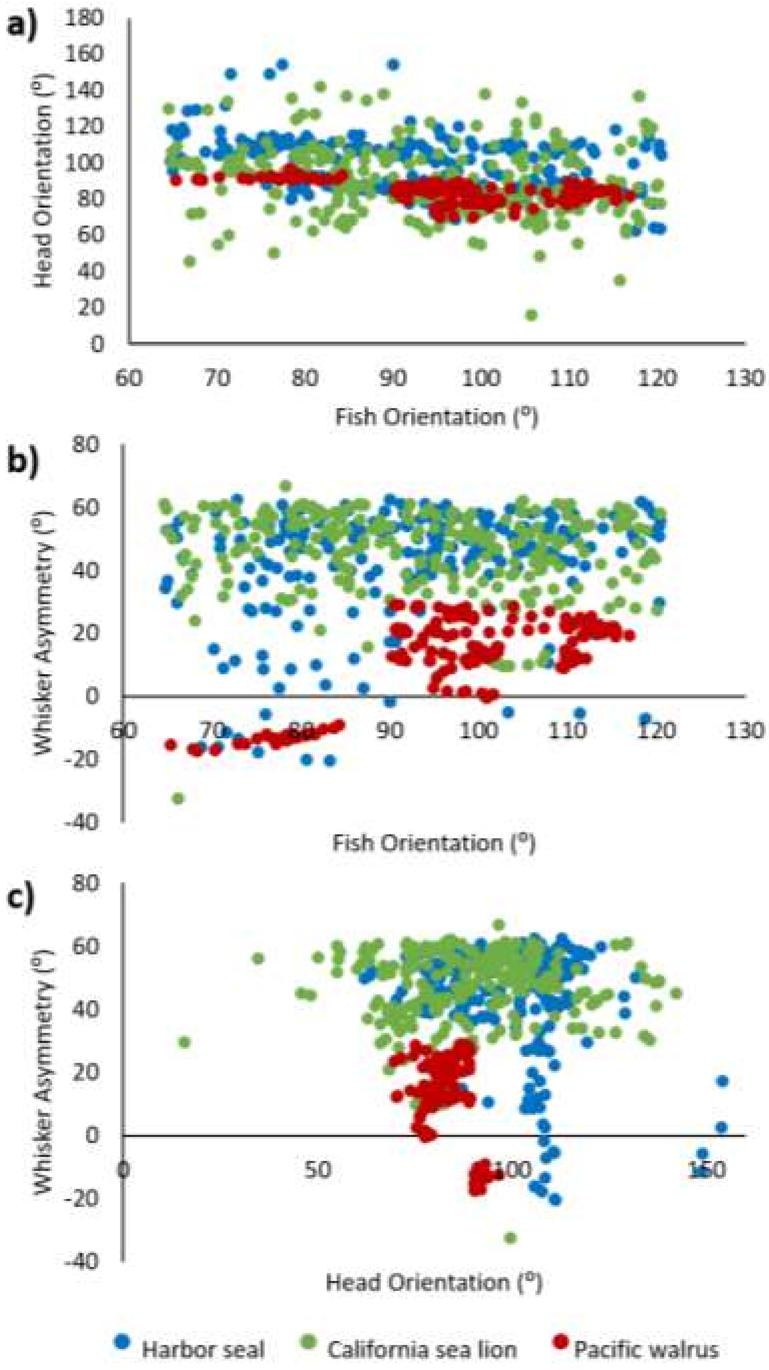

Fig. S2.1 Head, fish and whisker angles in response to fish sweeping for fish orientations of 65-120°: Scattergrams of per-frame angles across all tracked videos for:(a) Fish Orientation vs. Head Orientation, (b) Fish Orientation vs. Whisker Asymmetry, (c) Head Orientation vs. Whisker Asymmetry. Head orientation is correlated to the fish orientation in California sea lion and Pacific walrus (Spearman's rank Correlation: $p < 0.05$), but not Harbor seal, in panel a. Fish orientation and whisker asymmetry is correlated in California sea lion and Pacific walrus (Spearman's rank Correlation: $p < 0.05$), but not in Harbor seal in panel b. Whisker asymmetry and head orientation is correlated in Pacific walrus in panel c (Spearman's rank Correlation: $p < 0.05$), but not in California sea lion or Harbor seal

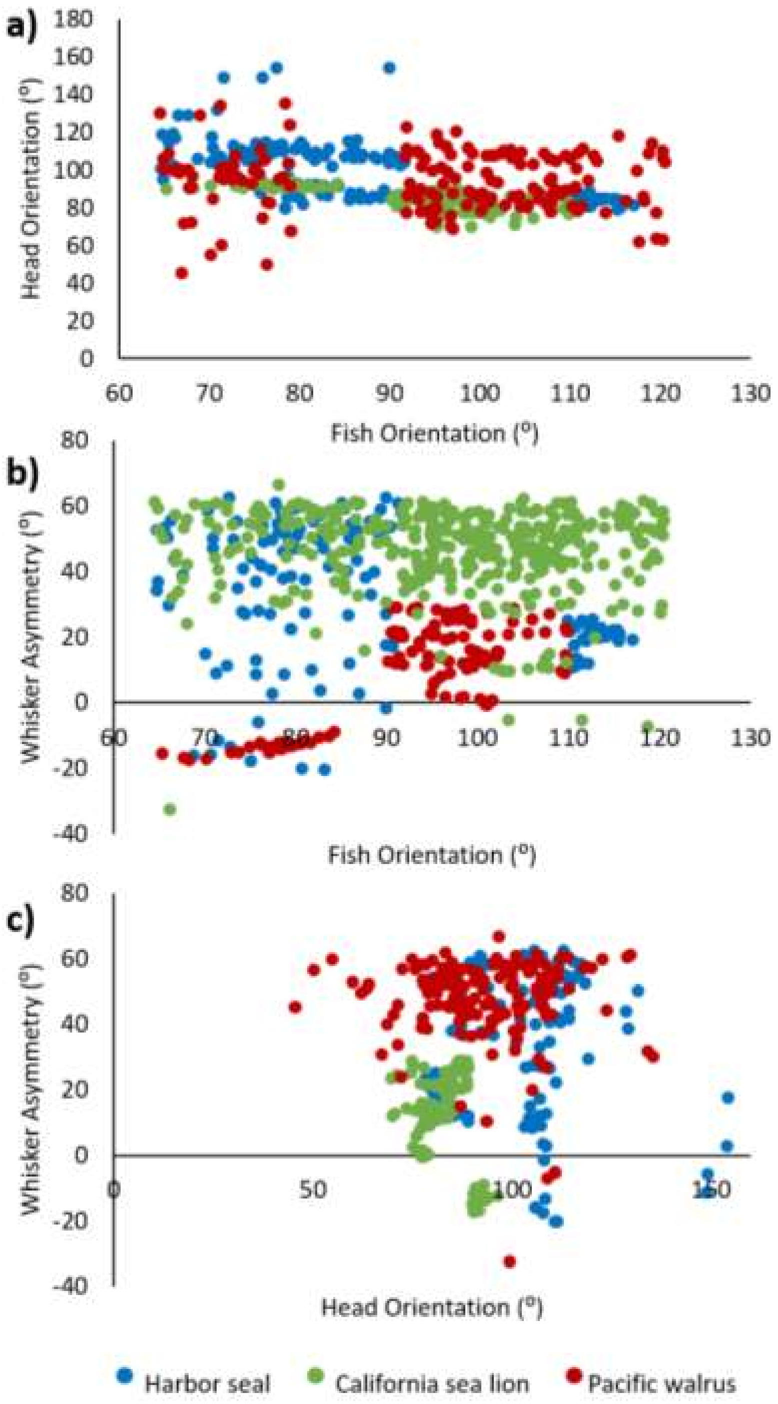

Fig. S2.2 Head, fish and whisker angles in response to fish sweeping for fish orientations of 65-120° and fish speeds of 0-0.3$^{o/s}$. Scattergrams of per-frame angles across all tracked videos for:(a) Fish Orientation vs. Head Orientation, (b) Fish Orientation vs. Whisker Asymmetry, (c) Head Orientation vs. Whisker Asymmetry. Head orientation is correlated to the fish orientation in all species (Spearman's rank Correlation: $p < 0.05$), in panel a. Fish orientation and whisker asymmetry is correlated in California sea lion and Pacific walrus (Spearman's rank Correlation: $p < 0.05$), but not in Harbor seal in panel b. Whisker asymmetry and head orientation is correlated in Pacific walrus in panel c (Spearman's rank Correlation: $p < 0.05$), but not in California sea lion or Harbor seal

Table S1: Corrrelation tests of Head, fish and whisker angles. The table shows correlation tests from all data and also controlling for fish movement by: : i) only including video frames with fish orientations of 65-120°; ii) only including video frames with fish orientations of 65-120° and controlling for fish speed using partial correlations; iii) only including video frames with fish orientations of 65-120° and fish speeds of 0-0.3^o/s. Significant correlations ($p<0.05$) are indicated with a tick, and correlation tests that are not significant ($p>0.05$) are indicated with a cross.

Species and Condition	Statistical Test	Fish Orientation and Whisker Asymmetry	Fish Orientation and Head Orientation	Head Orientation and Whisker Asymmetry
**California Sea lion**				
All data	Spearman's Rank	✓	✓	✓
fish orientation 65-120°	Spearman's Rank	✓	✓	**X**
fish orientation 65-120°, controlling for fish speed	Partial Correlation	✓	✓	✓
fish orientation 65-120°, fish speed 0-0.3^o/s	Spearman's Rank	✓	✓	**X**
**Harbor seal**				
All data	Spearman's Rank	**X**	✓	**X**
fish orientation 65-120°	Spearman's Rank	**X**	✓	**X**
fish orientation 65-120°, controlling for fish speed	Partial Correlation	**X**	✓	**X**
fish orientation 65-120°, fish speed 0-0.3^o/s	Spearman's Rank	**X**	**X**	**X**
**Pacific walrus**				
All data	Spearman's Rank	✓	✓	✓
fish orientation 65-120°	Spearman's Rank	✓	✓	✓
fish orientation 65-120°, controlling for fish speed	Partial Correlation	✓	✓	✓
fish orientation 65-120°, fish speed 0-0.3^o/s	Spearman's Rank	✓	✓	✓

Supplementary Material 3:

Examining the effect of eye sight on Pinniped whisker positions and movements

Whisker position and movement data were re-tested, comparing blind and sighted animals. Firstly the Harbor seals were tested with Wanda (who had cataracts in both eyes) compared to Ina and Pamina (both sighted animals), and there was no significant differences in whisker movement and position in terms of offset (Mann-Whitney U: U=81, p=0.683), amplitude (Mann-Whitney U: U=83, p=0.755), spread (Mann-Whitney U: U=65, p=0.236) or asymmetry (Mann-Whitney U: U=57, p=0114) between the blind and sighted Harbor seals. Secondly, the Pacific walruses were tested with Olga (who was considered by her trainers to be completely blind) compared to Rossita and Olivia (both sighted animals). There were also no significant differences in offset (Mann-Whitney U: U=0, p=0.200), amplitude (Mann-Whitney U: U=1, p=0.400), spread (Mann-Whitney U: U=2, p=0.800) and asymmetry (Mann-Whitney U: U=1, p=0.400) between the blind and sighted Pacific Walruses.

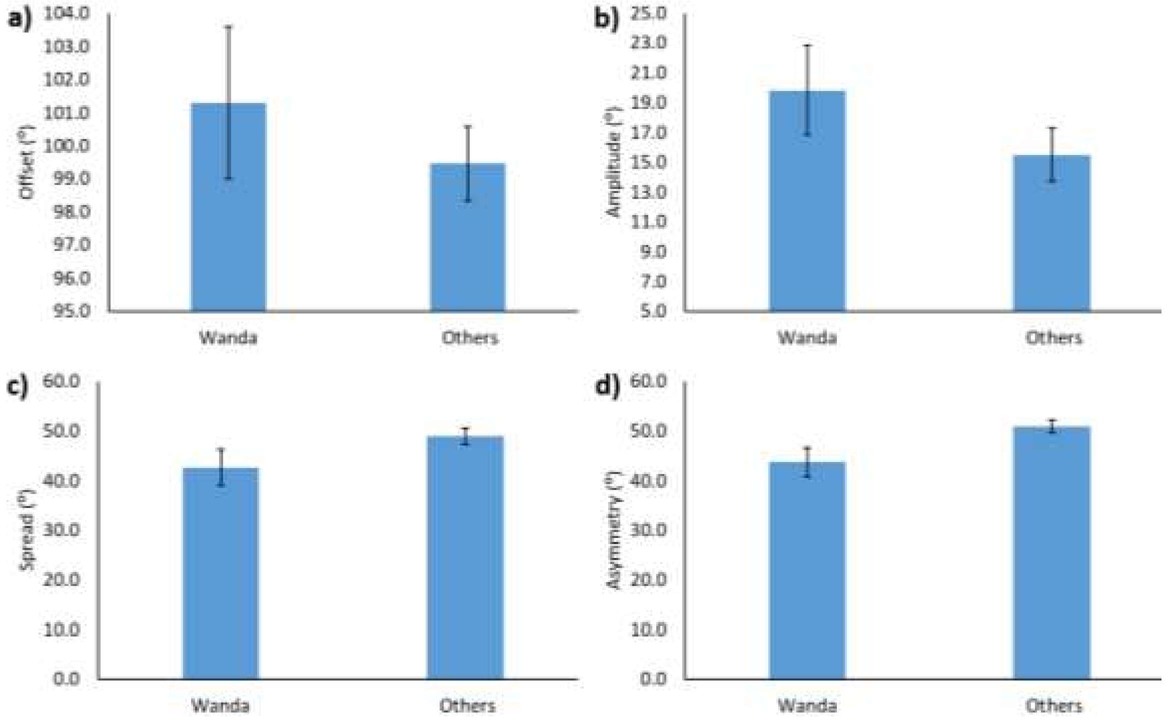

Fig. 3.1 Harbor seal whisker positions and movements, comparing Wanda (with cataracts in both eyes) to other sighted animals (Ina and Pamina): (a) offset, (b) amplitude, (c) spread and (d) show no significant differences between Wanda and the other sighted animals. All graphs show median values in degrees with error bars indicating upper and lower interquartile ranges

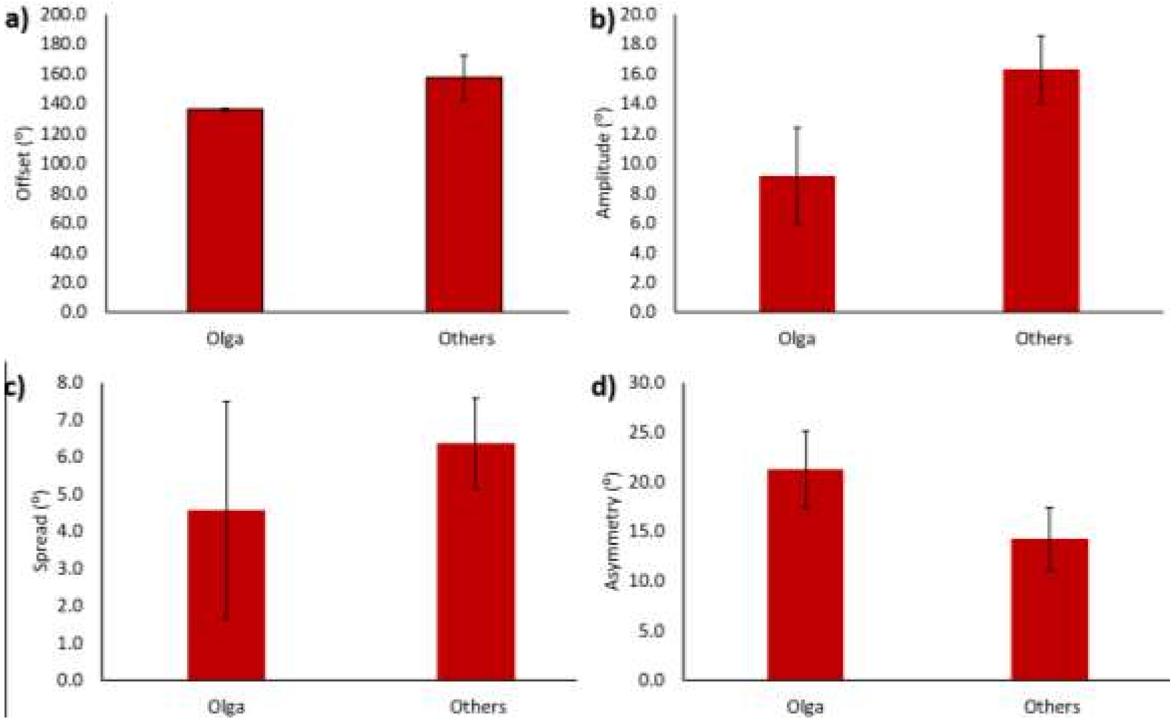

Fig. 3.2 Pacific walrus whisker positions and movements, comparing Olga (blind in both eyes) to other sighted animals (Rossita and Olivia): (a) offset, (b) amplitude, (c) spread and (d) show no significant differences between Olga and the other sighted animals. All graphs show median values in degrees with error bars indicating upper and lower interquartile ranges
